# Piora Hospitalar da Insuficiência Cardíaca: Podemos Prevê-la na Admissão?

**DOI:** 10.36660/abc.20230525

**Published:** 2023-08-28

**Authors:** Humberto Villacorta

**Affiliations:** 1 Universidade Federal Fluminense Niterói RJ Brasil Universidade Federal Fluminense, Niterói, RJ – Brasil

**Keywords:** Insuficiência Cardíaca, Descompensação Cardíaca, Prognóstico, Fatores de Risco, Admissão do Paciente

A insuficiência cardíaca aguda descompensada (ICDA) é um marcador de risco na trajetória do paciente com insuficiência cardíaca (IC). Pode ser a apresentação inicial (IC *de novo*) ou uma exacerbação aguda da IC crônica.^1^ A maioria dos pacientes com ICAD chega ao pronto-socorro (PS) com congestão e geralmente responde bem ao tratamento com diuréticos.^2^ No entanto, alguns pacientes de alto risco podem ter um curso complicado durante a hospitalização, incluindo piora intra-hospitalar da IC (PIC), definida como sinais e sintomas persistentes ou agravados que requerem uma escalada na terapia.^3^ A identificação desses pacientes é importante, pois apresentam maior risco de eventos intra-hospitalares e pós-alta.^4,5^

**Figura 1:**
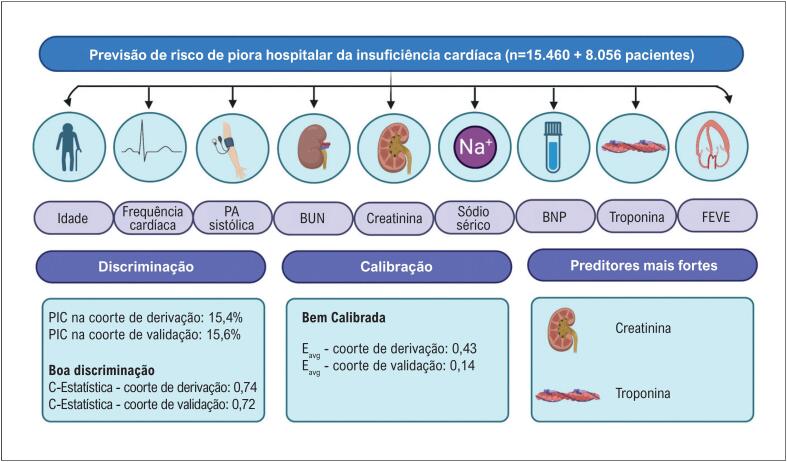
Síntese dos achados do modelo de risco ADHERE para predizer a PIC intra-hospitalar em pacientes internados com ICAD. O estudo incluiu 15.640 pacientes na coorte de derivação e 8.056 pacientes na coorte de validação. Nove variáveis foram incluídas no modelo final. PA: pressão arterial; BUN: nitrogênio ureico no sangue; BNP: peptídeo natriurético tipo B; Eavg: índice estatístico que utiliza a média como medida de tendência central para resumir as diferenças absolutas entre probabilidades preditas e observadas; PIC: piora da insuficiência cardíaca; FEVE: fração de ejeção do ventrículo esquerdo.

No início dos anos 2000, a ICDA começou a atrair a atenção de muitos pesquisadores na área de IC. Nesse período, um grande registro de pacientes com ICAD – o Registro ADHERE – foi criado nos Estados Unidos da América.^6^ O Registro ADHERE deu enormes contribuições ao campo da ICDA. Eles nos ensinaram sobre as características clínicas e os resultados desses pacientes, mas, mais importante, criaram regras de previsão para pacientes admitidos com ICAD. Eles inicialmente relataram uma análise de árvore de regressão, usando três variáveis simples para prever a mortalidade intra-hospitalar – nitrogênio ureico no sangue (BUN), creatinina e pressão arterial sistólica.^7^ Dois anos depois, relataram o valor do peptídeo natriurético tipo B (BNP) na predição de mortalidade intra-hospitalar.^8^ Em 2016, eles desenvolveram e validaram um modelo para prever PIC intra-hospitalar, que incluiu variáveis clínicas (idade, frequência cardíaca, pressão arterial sistólica), exames laboratoriais (BUN, creatinina, sódio sérico), biomarcadores (BNP e troponina) e fração de ejeção do ventrículo esquerdo (FEVE).^3^ Seu modelo teve boa discriminação, com c-estatísticas de 0,74 e 0,72 para as coortes de derivação e validação, respectivamente, conforme mostrado na Figura 1. No entanto, a discriminação foi modesta na coorte de validação externa (estudo ASCEND-HF) (c-estatística 0,63).^3^

Nesta edição dos Arquivos Brasileiros de Cardiologia, Bernardes et al.^9^ apresentam um estudo abordando a questão da predição de PIC intra-hospitalar em uma população brasileira.^9^ Eles aplicaram o modelo de risco ADHERE a 890 pacientes internados por ICAD. A PIC intra-hospitalar ocorreu em 40,8% de toda a população. O modelo ADHERE identificou a maioria dos pacientes sem risco de PIC. No entanto, a discriminação foi modesta em comparação com a coorte de derivação original usada no Registro ADHERE, com área sob a curva de 0,66 e sensibilidade e especificidade de 66,9% e 55,2%, respectivamente.

As diferenças nas duas populações podem ser responsáveis pelo menor poder discriminatório na coorte brasileira. A população do estudo de Bernardes parece ter IC mais grave do que a população do ADHERE. A taxa de PIC foi muito maior na coorte brasileira (40,8% vs 15,4%). Da mesma forma, os pacientes do estudo brasileiro apresentaram menor pressão arterial e menor fração de ejeção (metade dos pacientes do registro ADHERE apresentou FEVE >40%). O artigo não mostra o desempenho individual dos componentes do escore, e gostaria de saber como o BNP se saiu no presente estudo. Foi demonstrado que o BNP na admissão prediz a mortalidade intra-hospitalar, mas faltam dados sobre PIC.^8,10^

Este é o primeiro estudo a avaliar o valor de um escore para prever PIC intra-hospitalar no Brasil, e parabenizamos os autores por trazê-lo à tona. Apesar das limitações acima, o escore ADHERE pode ajudar os médicos a identificarem pacientes de alto risco com ICAD no pronto-socorro. No entanto, como os autores apontaram no artigo, não deve ser usado como uma ferramenta única para prever PIC intra-hospitalar.
