# Supplementary material for: Piora Hospitalar da Insuficiência Cardíaca: Podemos Prevê-la na Admissão?
Source: Arq Bras Cardiol. 2023 Aug 28;120(8):e20230525. [Article in Portuguese] doi: 10.36660/abc.20230525 (PMC10519240; doi:10.36660/abc.20230525)
Supplement: Translation

# Translation: In-Hospital Worsening Heart Failure: Can We Predict It At Admission?

**Authors:** Humberto Villacorta

**Affiliations:** 1 Niterói RJ Brazil Universidade Federal Fluminense, Niterói, RJ – Brazil

**Keywords:** Heart Failure, Heart Decompensation, Prognosis, Risk Factors, Patient Admission

Acute decompensated heart failure (ADHF) is a risk marker in the patient's heart failure (HF) journey. It may be the initial presentation (de novo HF) or an acute exacerbation of chronic HF.^1^ Most patients with ADHF present to the emergency department (ED) with congestion and usually respond well to treatment with diuretics.^2^ However, some high-risk patients may have a complicated course during hospitalization, including in-hospital worsening HF (WHF), defined as persistent or worsening signs and symptoms requiring an escalation in therapy.^3^ Identifying such patients is important since they are at greater risk of in-hospital and post-discharge events.^4,5^

In the early 2000s, ADHF began to attract the attention of many researchers in the field of HF. In that period, a large registry of patients with ADHF – the ADHERE Registry – was set up in the United States of America.^6^ The ADHERE Registry gave enormous contributions to the field of ADHF. They taught us about these patients’ clinical characteristics and outcomes, but more importantly, they created prediction rules for patients admitted with ADHF. They initially reported a regression tree analysis, using three simple variables to predict in-hospital mortality – blood urea nitrogen (BUN), creatinine, and systolic blood pressure.^7^ Two years later, they reported the value of B-type natriuretic peptide (BNP) in predicting in-hospital mortality.^8^ In 2016 they developed and validated a model to predict in-hospital WHF, which included clinical variables (age, heart rate, systolic blood pressure), laboratory tests (BUN, creatinine, serum sodium), biomarkers (BNP and troponin), and the left ventricle ejection fraction (LVEF).^3^ Their model had good discrimination, with c-statistics of 0.74 and 0.72 for the derivation and validation cohorts, respectively, as shown in Figure 1. Nevertheless, discrimination was modest in the external validation cohort (ASCEND-HF study) (c-statistic 0.63).^3^

**Figure 1:** Summary of the findings of the ADHERE risk model to predict in-hospital WHF in patients admitted with ADHF. The study included 15640 patients in the derivation cohort and 8056 patients in the validation cohort. Nine variables were included in the final model. BP: blood pressure; BUN: blood urea nitrogen; BNP: B-type natriuretic peptide; E_avg:_ statistical index that uses the mean as the measure of central tendency for summarizing the absolute differences in between predicted and observed probabilities; WHF: worsening heart failure; LVEF: left ventricle ejection fraction.

In this *Arquivos Brasileiros de Cardiologia* issue, Bernardes et al. present a study addressing the issue of predicting in-hospital WHF in a Brazilian population.^9^ They applied the ADHERE risk model to 890 patients admitted for ADHF. In-hospital WHF occurred in 40.8% of the entire population. The ADHERE model identified the majority of patients not at risk for WHF. However, the discrimination was modest compared to the original derivation cohort used in the ADHERE Registry, with an area under the curve of 0.66 and sensitivity and specificity of 66.9% and 55.2%, respectively.

Differences in the two populations may account for the lower discriminatory power in the Brazilian cohort. The population in Bernardes’ study seems to have more severe HF than the ADHERE population. The rate of WHF was much higher in the Brazilian cohort (40.8% vs 15.4%). Likewise, patients in the Brazilian study had lower blood pressure and lower ejection fraction (half of the patients in the ADHERE registry had LVEF >40%). The article does not show the individual performance of the score components, and I wonder how BNP performed in the present study. Admission BNP has been shown to predict in-hospital mortality, but data on WHF is lacking.^8,10^

This is the first study to assess the value of a score to predict in-hospital WHF in Brazil, and we congratulate the authors for bringing this up. Despite the above limitations, the ADHERE score may help physicians identify high-risk patients with ADHF in the ED. However, as the authors pointed out in the article, it should not be used as a single tool to predict in-hospital WHF.
